# Rainfall extremes: Toward reconciliation after the battle of distributions

**DOI:** 10.1002/2013WR014211

**Published:** 2014-01-15

**Authors:** Francesco Serinaldi, Chris G Kilsby

**Affiliations:** 1 School of Civil Engineering and Geosciences, Newcastle UniversityNewcastle Upon Tyne, UK; 2 Willis Research NetworkLondon, UK

**Keywords:** extreme events, precipitation, time series analysis, peak-over-threshold analysis, heavy tail behavior

## Abstract

[1] This study attempts to reconcile the conflicting results reported in the literature concerning the behavior of peak-over-threshold (POT) daily rainfall extremes and their distribution. By using two worldwide data sets, the impact of threshold selection and record length on the upper tail behavior of POT observations is investigated. The rainfall process is studied within the framework of generalized Pareto (GP) exceedances according to the classical extreme value theory (EVT), with particular attention paid to the study of the GP shape parameter, which controls the heaviness of the upper tail of the GP distribution. A twofold effect is recognized. First, as the threshold decreases, and nonextreme values are progressively incorporated in the POT samples, the variance of the GP shape parameter reduces and the mean converges to positive values denoting a tendency to heavy tail behavior. Simultaneously, the EVT asymptotic hypotheses are less and less realistic, and the GP asymptote tends to be replaced by the Weibull penultimate asymptote whose upper tail is exponential but apparently heavy. Second, for a fixed high threshold, the variance of the GP shape parameter reduces as the record length (number of years) increases, and the mean values tend to be positive, thus denoting again the prevalence of heavy tail behavior. In both cases, i.e., threshold selection and record length effect, the heaviness of the tail may be ascribed to mechanisms such as the blend of extreme and nonextreme values, and fluctuations of the parent distributions. It is shown how these results provide a link between previous studies and pave the way for more comprehensive analyses which merge empirical, theoretical, and operational points of view. This study also provides several ancillary results, such as a set of formulae to correct the bias of the GP shape parameter estimates due to short record lengths accounting for uncertainty, thus avoiding systematic underestimation of extremes which results from the analysis of short time series.

**Citation:** Serinaldi, F., and C. G. Kilsby (2014), Rainfall extremes: Toward reconciliation after the battle of distributions, *Water Resour. Res*., 50, 336–352, doi:10.1002/2013WR014211.

## 1. Introduction

[2] The history of extreme value theory (EVT) in its present formalization and its application to hydrologic analyses is well rooted in an extensive literature dating back to the 1940s. Focusing on univariate frequency analysis and referring to *Papalexiou and Koutsoyiannis* [2013] for a recent overview of the history of EVT, this theory deals essentially with the asymptotic distributional behavior of two types of data, namely, the so-called “block maxima” (BMs) and “peaks over threshold” (POTs). The first type refers to the maximum values extracted from blocks (subsets) of observations, whereas the second type to observations that exceed a given threshold. As the size of the blocks approaches infinite, the Fisher-Tippett-Gnedenko theorem [*Fisher and Tippett*, 1928; *Gnedenko*, 1943] shows that the distribution of BM converges to three types of extreme value distributions (Gumbel, Fréchet, and reverse Weibull) which can be described by the unified von Mises-Jenkinson parameterization [*Jenkinson*, 1955] of the so-called generalized extreme value (GEV) distribution [e.g., *Coles*, 2001, pp. 47–48]


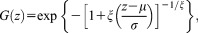
(1)

where 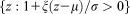, 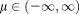 is a location parameter, 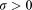 is a scale parameter, and 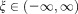 is a shape parameter. On the other hand, as the threshold increases, the Pickands-Balkema-de Haan theorem [*Pickands III*, 1975; *Balkema and de Haan*, 1974] establishes that the distribution of POT 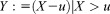 converges to the so-called generalized Pareto (GP) distribution [e.g., *Coles*, 2001, pp. 75–76]


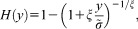
(2)

where 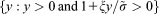 and 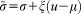.

[3] EVT establishes a link between these two distributions (and the underlying types of data). Indeed, if block maxima have approximate distribution GEV, then threshold excesses have a corresponding approximate distribution within the GP family [e.g., *Coles*, 2001, p. 75] and vice versa GEV parameterization can be obtained from GP under suitable conditions (i.e., Poisson distribution for the occurrence frequency of POT) [e.g., *Goda*, 2011]. In other words, the parameters of the GP distribution of threshold excesses are uniquely determined by those of the associated GEV distribution of block maxima [e.g., *Coles*, 2001, p. 75]. In particular, the shape parameter *ξ* of GP is equal to that of the corresponding GEV distribution. The value of *ξ* characterizes the upper tail behavior of GP and GEV: if 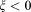 the distribution of POT and BM has an upper bound; if 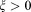 the distribution has no upper limit and is denoted as subexponential or heavy-tailed as the upper tail of the density function decays as a power law, i.e., more slowly than an exponential distribution [*Embrechts et al*., 1997, p. 39]. If 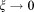, GEV and GP converge to Gumbel and exponential distributions, respectively, viz, distributions with exponential tails.

[4] Since heavy tail behavior implies a probability of extreme events higher than that returned by exponentially tailed distributions, and this impacts on the design values to be used in the applications, the correct assessment of *ξ* value plays a key role in hydrological frequency analysis. This has stimulated an extensive investigation of the upper tail behavior of hydrological variables [e.g., *Katz et al*., 2002] such as a streamflow records [e.g., *De Michele and Rosso*, 2001; *Bernardara et al*., 2008] and rainfall measurements [e.g., *Salvadori and De Michele*, 2001; *Beguería*, 2005; *De Michele and Salvadori*, 2005; *Deidda and Puliga*, 2006] just to mention a few recent examples. Focusing on rainfall, many studies investigated extreme values in several geographic areas and different time scales through EVT analyses from different perspectives. Broadly speaking, this literature can be classified according to the degree of attention to empirical or theoretical aspects, going from studies more focused on the examination of rainfall extremes in particular areas by well-established EVT techniques [e.g., *Li et al*., 2005; *Beguería and Vicente-Serrano*, 2006; *Khan et al*., 2007; *Vicente-Serrano et al*., 2009; *Villarini et al*., [Bibr b71],[Bibr b72]] to studies that propose inferential techniques and are therefore more focused on mathematical/statistical aspects [e.g., *Rasmussen and Rosbjerg*, 1991; *Wang*, 1991; *Salvadori*, 2003; *Beguería*, 2005; *Ashkar and Nwentsa Tatsambon*, 2007; *Deidda*, 2007; *Willems et al*., 2007; *Clauset et al*., 2009; *Deidda and Puliga*, 2009; *Langousis et al*., 2013]. Moreover, beside techniques devised for independent and identically distributed (i.i.d.) data, there is much recent interest in nonstationary POT models [e.g., *Khaliq et al*., 2006; *Sugahara et al*., 2009; *Acero et al*., 2011; *Beguería et al*., 2011; *Aryal et al*., 2009; *Roth et al*., 2012; *Tramblay et al*., 2013].

[5] Among the different perspectives applied to the study of POT rainfall, there is a line of inquiry that aims to better understand the link between EVT and empirical results accounting for the limits of the analyzed data sets and the fluctuations of the physical processes that generate the rainfall records. Since extreme events are rare by definition, EVT asymptotic conditions (which hold for infinite samples) are far from being even approximately valid for finite samples, and an ill-founded confidence on the validity of EVT assumptions can easily result in errors in the inference outcomes. This type of study is therefore a fundamental requirement in order to correctly interpret the outputs of EVT analyses, assess their reliability accounting for the intrinsic lack of data, and avoid misleading conclusions.

[6] In this respect, *Koutsoyiannis* [2004a] provided a theoretical critique of the validity of the two oversimplifying assumptions that are behind the use of the Gumbel distribution (i.e., the parent observations of BM can be represented as i.i.d. random variables, and the parent distribution belongs to the domain of attraction of the Gumbel family) and showed that small and realistic departures for these hypotheses (e.g., fluctuations of the parameters of the parent distribution) result in convergence to GEV (with *ξ* values corresponding to heavy-tailed Fréchet-like asymptote) rather than to the exponentially tailed Gumbel distribution. Moreover, the small size of the samples, usually less than 50 annual maxima (AMs), tends to hide the heavy tail behavior, thus leading to selection of the Gumbel option even though the true distribution is GEV. *Koutsoyiannis* [2004b] further studied the effect of the sample size by analyzing 169 rainfall time series worldwide that cover 100–154 years of record. The analysis was performed both on the series of AM of daily rainfall and on the series of POT, chosen so that the number of values corresponds to the number of years of the record. *Koutsoyiannis* [2004b] introduced the hypothesis that the shape parameter is constant (∼0.15) and independent of the geographic regions by ascribing the at-site variability to the sampling uncertainty. Under this assumption, he showed that GEV distribution with 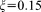 provides a description of daily rainfall AM more realistic than its two-parameter special cases (i.e., Gumbel and Fréchet). *Papalexiou and Koutsoyiannis* [2013] further investigated these empirical results by analyzing 15,137 worldwide rainfall series, with length varying from 40 to 163 years. Focusing on the AMs, *Papalexiou and Koutsoyiannis* [2013] studied the effect of the sample size on the estimation of the GEV shape parameter and concluded that there is empirical evidence that the GEV shape parameter is not constant (as previously hypothesized by *Koutsoyiannis* [2004b]) but follows approximately a Gaussian distribution with mean ∼0.114 and standard deviation ∼0.045 as the sample size tends to infinite. They also suggested a simple linear transformation which corrects the bias caused by the finite sample size and preserves the spatial patterns of the *ξ* estimates related to the different climatic areas of the globe (see maps in Figures 12 and 13 of *Papalexiou and Koutsoyiannis* [2013]). Accounting for the sampling bias, it follows that the true GEV shape parameter is always positive, thus indicating that the unbounded Fréchet distribution with power-law upper tail describes the extreme daily rainfall values more accurately than the two other EVT asymptotes (i.e., reversed Weibull and Gumbel).

[7] *Papalexiou et al*. [2013] used the same worldwide rainfall data set to study the performance of four distributions (Pareto, Gamma, Weibull, and lognormal) to fit the POT selected by six different methods and concluded that Pareto outperforms the other distributions, thus confirming the suitability of heavy-tailed distributions for extreme rainfall values. In addition, they also found that the mode of the Pareto shape parameter is 0.134, which is not far from the GEV shape parameter for AM, even though the POT analysis was not performed following the standard methods of EVT and exploiting the duality between GEV and GP [e.g., *Coles*, 2001].

[8] In this context, by applying the large deviation theory and multifractal beta-lognormal multiplicative random cascade models, *Veneziano et al*. [2009] have obtained asymptotic results different from the classical EVT, indicating that (a) the value of the GEV (and GP) shape parameter is always higher than that provided by the classical EVT, (b) this value depends on the aggregation scale, and (c) *ξ* does not depend on the tail of the rainfall distribution but on the main body of distribution as previously argued by *Klemeš* [2000]. *Veneziano et al*. [2009] also proposed a near-universal relationship to estimate the GEV (GP) shape parameter as a function of the aggregation time scale. This relationship returns a value very close to that found by *Koutsoyiannis* [2004b] for long spanning daily rainfall series (see also *Veneziano and Yoon* [2013] for further developments).

[9] *Papalexiou et al*. [2013] found that the effect of a limited sample size can hide the subexponential behavior of POT similarly to BM. However, they studied POT without focusing specifically on this problem. Therefore, in this study, a subset of the database analyzed by *Papalexiou and Koutsoyiannis* [2013] is used to investigate the behavior of POT within the GP framework in order to show the POT GP-based counterpart of the annual BM GEV-based analysis presented by *Papalexiou and Koutsoyiannis* [2013] and provide further empirical evidence of the hiding effect of sample size.

[10] In the remainder of this paper, we first introduce the data set and the methodology in section and then present and discuss the results in sections. Concluding remarks are reported in section.

## 2. Materials and Methods

### 2.1 Data Set

**Figure 1:**
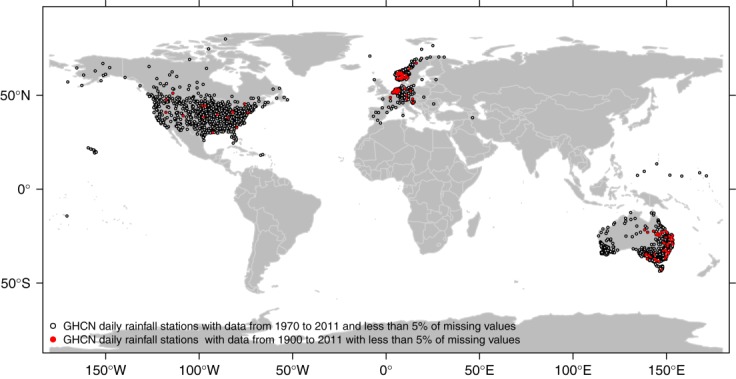
Location of GHCN rainfall records used in the analyses.

[11] The data set used in the analyses is a subset of the Global Historical Climatology Network (GHCN) data set available at the web site (www.ncdc.noaa.gov/oa/climate/ghcn-daily). GHCN-Daily data set contains daily data from over 80,000 surface stations worldwide, about two thirds of which are for precipitation only [*Menne et al*., 2012]. The data set was retrieved and handled by the R contributed package GhcnDaily [*Mosher*, 2012]. Since the POT analysis requires possibly complete time series, only a limited subset of the available time series was retained. In more detail, two subsets of data are selected: (1) rainfall series spanning from 1970 to 2011 with less than 5% of missing values and (2) rainfall series spanning from 1900 to 2011 with less than 5% of missing values (Figure 1). The additional selection criteria based on the quality flags used by *Papalexiou et al*. [2013] were also applied along with a check of the random distribution of missing values and a visual check of each time series and its empirical distribution function to detect possible macroscopic inconsistencies related to measurement errors. Our selection differs from that of *Papalexiou et al*. [2013] as we decided to use only time series covering the same periods (i.e., 1970–2011 and 1900–2011) so that the series reflect the worldwide climate conditions over homogeneous time windows. Obviously, this criterion is more restrictive than the 50 year minimum length used by *Papalexiou et al*. [2013] and resulted in a smaller number of time series, namely, 1898 for the shortest series and 113 for the longest ones.

### 2.2 Methodology

[12] The two data sets described in the previous section are used to study the behavior of the POT rainfall values. We tested (1) the significance of lag-1 correlation for two subsequent values by the Kendall correlation coefficient (K-ACF), (2) possible monotonic trends by the Mann-Kendall (M-K) test, and (3) distributional hypotheses by goodness of fit and ad hoc diagnostics. In more detail, the suitability of GP distribution for POT values is assessed by four goodness of fit tests, namely, Kolmogorov-Smirnov (K-S), Cramer-von Mises (C-vM), Anderson-Darling (A-D), and the Pearson product moment correlation coefficient on the *P-P* plots (PPMCC) [e.g., *Filliben*, 1975; *Laio*, 2004; *Kottegoda and Rosso*, 2008]. All the statistical tests are performed at the 5% significance level.

[13] It should be noted that the application of K-ACF and M-K tests is a fundamental step as the presence of possible temporal dependence and monotonic trends can affect and bias the outcome of the goodness of fit tests, which rely on the hypothesis of independent observations. Moreover, the results can be also influenced by the presence of spatial correlation [e.g., *Douglas et al*., 2000; *Daniel et al*., 2012; *Guerreiro et al*., 2014]. However, even though some relationships can exist between POT events occurring in nearby sites, the selected POT events are rarely simultaneous and the mutual distances between the sites allow us to assume the spatial independence as a reasonable assumption.

[14] In order to complement the previous works by *Papalexiou et al*. [2013] and *Papalexiou and Koutsoyiannis* [2013], where the GP parameters are estimated by L-moments [*Hosking*, 1990] and mean square error minimization, we used the maximum likelihood (ML) approach for two reasons: (1) to show that the coherence between our results and previous findings is reasonably independent of the estimation method and (2) to set the discussion within the most popular framework used in stationary and nonstationary POT frequency analysis, thus making the results comparable with those reported in a large body of literature relying on ML and related software.

[15] As an additional exploratory tool, we also use the so-called maximum likelihood multiple threshold method (MTM) [*Deidda*, 2010]. This approach relies on the expression of the mixed (discrete-continuous) distributions suitable for zero-inflated data such as daily rainfall [e.g., *Kedem et al*., 1990], 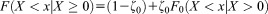, where 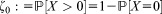 and *F*_0_ is the distribution of strictly positive values. Assuming that *F*_0_ is GP the mixed distribution *F* specializes as follows:


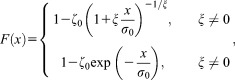
(3)

where 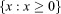, 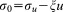, and 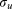 is the scale parameter corresponding with a generic threshold value *u*. Unlike *F*_0_, the mixed distribution in equation 3 allows the development of a hierarchical estimation method (MTM) [*Deidda*, 2010] that enables the calibration of a GP distribution that fits the data over a proper range of threshold values, thus smoothing out the fluctuations of *ξ* resulting from the choice of a single threshold.

[16] The 1970–2011 data set is used to explore how the outcome of the tests varies with different values of the threshold corresponding to 10 different rainfall percentiles (including zeros) ranging from 95% to 99.5% by 0.5% steps. This allows us to detect a possibly unique threshold generally valid for daily rainfall POT selection and analysis over a wide data set. Since the sample size of the selected POT values decreases as the threshold increases, the variability of the GP shape parameter over the range of thresholds is also studied. Based on the results corresponding to the 1970–2011 data set (presented in section), the same analysis is repeated for the 1900–2011 data set by fixing the threshold value at the 98th percentile and varying the sample size by extracting subsamples with different lengths from 10 to 110 years by 5 year steps. In this case, the variability of the GP shape parameter related to the sample size does not depend on the threshold selection but on the temporal extension of the available time series.

[17] Thus, the 1970–2011 sample is used to explore the effect on *ξ* of varying the number of events by keeping the record length unchanged, whereas the 1900–2011 sample is used to study the variability associated with the temporal fluctuations of the POT process for a fixed percentile threshold. The analyses are performed on a seasonal basis assuming a 6 month delay between the sites located in the northern and southern hemispheres. Namely, we distinguish four seasons such that the winter comprises December, January, and February in the northern hemisphere and June, July, and August in the southern hemisphere. Spring, summer, and autumn are defined similarly. Obviously, the 3 month periods must be considered as pseudo-seasons because of the large variability of the local climatology in the worldwide data set. Deseasonalization procedures were not considered to avoid possible artifacts that may affect the results.

## 3. Analysis of 1970–2011 Data

**Figure 2:**
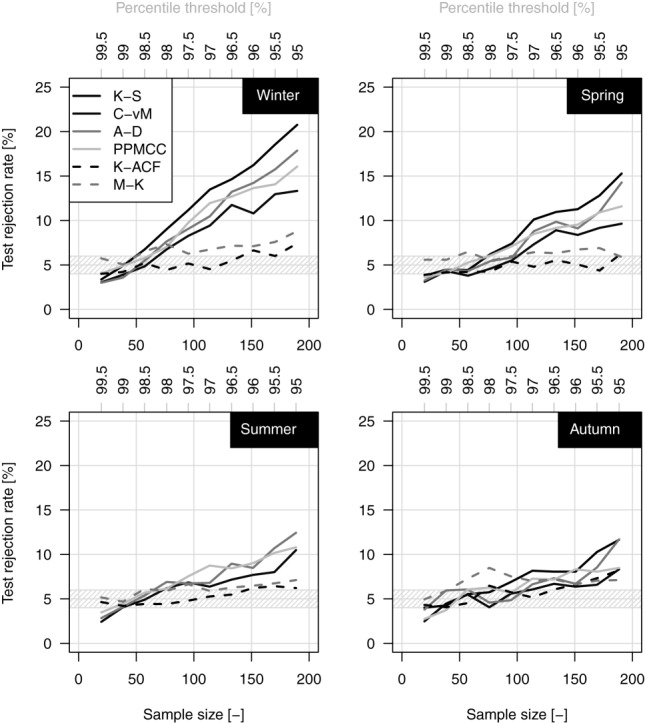
Percentage of rejection of the null hypotheses (“K-ACF = 0,” “no monotonic trend,” and “data from GP distribution”) for the 1970–2011 sample.

[18] As mentioned in the previous section, we evaluate the suitability of GP hypothesis for the POT values over all the 10 percentile thresholds by K-S, C-vM, A-D, and PPMCC goodness of fit tests. Additionally, the sequences of exceedances are tested for serial correlation by K-ACF and for possible monotonic trends by M-K test. Results are reported in Figure 2 in terms of percentage of rejection of the null hypotheses (i.e., “data from a GP distribution,” “K-ACF = 0,” and “no monotonic trend”) out of 1898 cases (i.e., the number of time series) for each test and each season. In this multiple testing exercise, under the hypothesis that the series is spatially uncorrelated, the number of failures to reject the null hypotheses when this is true follows a binomial distribution with expectation 95 (i.e., 5% of 1898) and 95% prediction interval (77,114), i.e., (4%, 6%).

[19] Figure 2 shows that the percentage of rejection of the “K-ACF = 0” hypothesis is close to the nominal value for all the thresholds, meaning that there is not evidence for time correlation between subsequent POT values apart from the expected random fluctuations. The percentage of rejection of M-K test is close to the expected nominal value (5%) for spring and summer, whereas a slight overrejection emerges for autumn and winter, denoting that possible monotonic trends are detected by M-K test slightly more frequently than expected, but no more than 7–8% of time series. The comparison between the K-ACF and M-K results indicates that the M-K results do not depend on the possible effect of temporal correlation.

[20] The percentage of rejection of the GP hypothesis is close to the nominal value only for thresholds around the 98th percentile (ranging from the 97.5th to 98.5th depending on the season and the specific goodness of fit test) corresponding to an average value of the sample size close to 1.8 times the number of record years (i.e., ∼75–76 events for each season). In other words, if we select more than two events per year (on a seasonal basis), the hypothesis of GP distribution is rejected in more cases than expected by chance. This is more evident in winter, in which the percentage of rejection reaches the 15–20% for the lowest thresholds based on the used tests. The overrejection is less evident for the other seasons and stands below or close to 10%. However, even though the percentage of rejection is higher than the nominal 5%, this does not mean that the fitting is unacceptable. On the contrary, a visual check (figures not shown) shows that GP generally fits data rather well. The overrejection can be ascribed to several causes such as the increasing power of the tests as the sample increases (thus leading to frequently reject the null hypothesis because of small discrepancies) [*Herr and Krzysztofowicz*, 2005; *Serinaldi*, 2009] and departures from the GP model related, for instance, to measurement approximations and errors [e.g., *Deidda and Puliga*, 2006] or actual inadequacy of GP to model rainfall as the threshold decreases. However, we shall return to the cause of this overrejection after studying the variability of the shape parameter *ξ* with the threshold selection.

**Figure 3:**
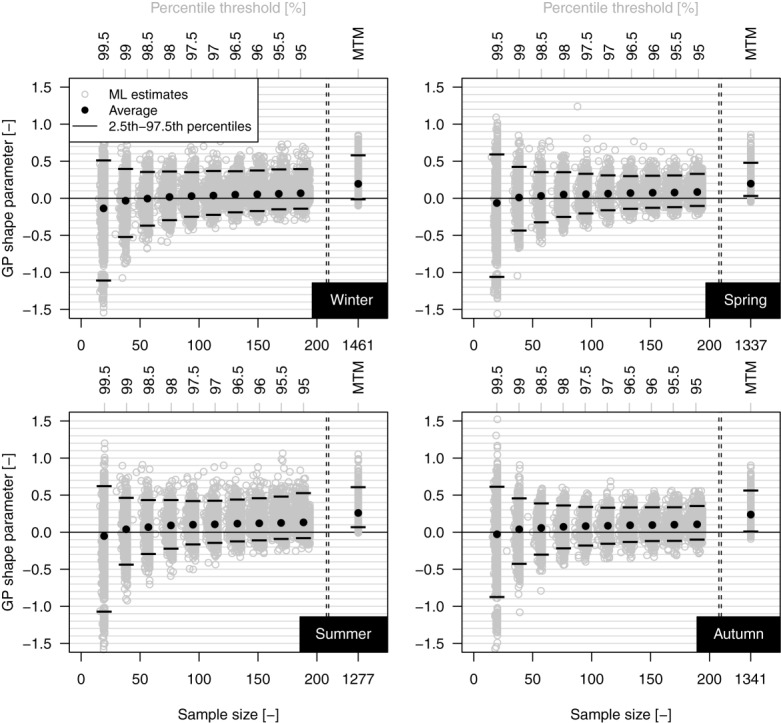
GP shape parameter versus sample size (percentile threshold) for the 1970–2011 sample.

[21] As mentioned in section, *ξ* controls the shape of the upper tail, discriminating between bounded, exponential, and power-law decay. In this respect, Figure 3 shows the *ξ* values along with their average and the 2.5th and 97.5th percentiles for each threshold value and season. The variability of *ξ* decreases as the threshold decreases and the corresponding sample size increases, whereas the average value of *ξ* increases and tends to stabilize around a positive value. These diagrams look like Figure 9a reported by *Papalexiou and Koutsoyiannis* [2013]; however, they differ because the varying sample size depends on the choice of the threshold rather than on the length of the time series for a fixed threshold (which is the case discussed in the next section by using the 1900–2011 data set).

[22] Both the *ξ* average and the variability tend to limiting values (if any) defined by the lowest possible threshold corresponding with zero rainfall. However, reducing the threshold value under the optimal range of values for which the EVT assumptions are deemed approximately valid for operational purposes, nonextreme rainfall values are progressively incorporated in the POT sample, and fitting a J-shaped GP distribution is questionable as strictly positive daily rainfall can also exhibit a bell-shaped distribution [*Papalexiou and Koutsoyiannis*, 2012]. Moreover, the choice of the POT threshold is always critical (not only for GP) because different threshold values often return different parameter estimates even if the sample is drawn from a known distribution. Therefore, instead of studying the limiting behavior of the *ξ* parameter corresponding to a “virtual” GP distribution working and fitted on 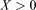, we use an alternative approach, viz, MTM. Since MTM relies on multiple ML estimates and exploits the information held in the middle and upper part of the distribution, the resulting MTM-based GP distribution can be seen as an approximation of the “virtual” limiting GP, which is however obtained without including the smallest rainfall values. Therefore, we can assume the MTM estimate of *ξ* as representative of the above mentioned limiting value when the threshold tends to zero under the hypothesis that the GP behavior characterizes 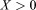. It should be noted that this does not mean that GP is deemed an optimal model for 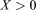. Actually, we are not interested in the quality of fit in the lower tail but in defining which is the prevalent sign of *ξ* (positive or negative) for a range of middle-high thresholds, smoothing out the fluctuations resulting from the choice of a single threshold.

**Figure 4:**
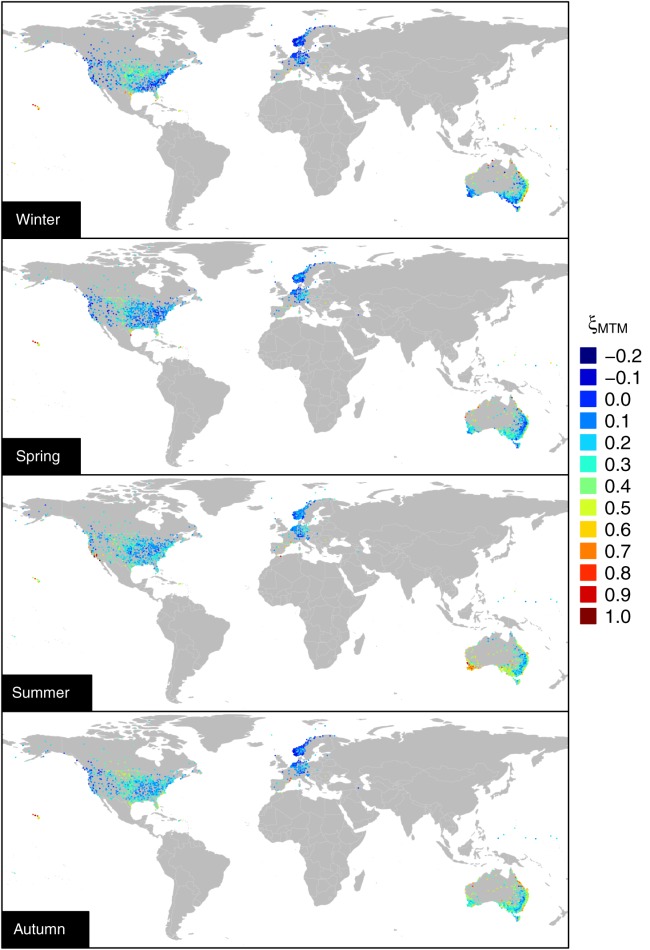
Spatial pattern of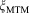 (see supporting information for local maps).

[23] The MTM estimates of *ξ* are shown in the right side of each plot of Figure 3. Almost all values are positive with a percentage of negative values going from 0.05% (1 series out of 1898) in summer to 3.58% (68 series out of 1898) in winter and an average value close to 0.2. Therefore, a subexponential behavior prevails in the middle and upper part of the daily rainfall distribution. In other words, provided that the “J” shape (with positive, negative, or null curvature in a log-linear plane) characterizes the probability density function of a left-truncated rainfall sample and GP can mimic it according to the sign of *ξ*, 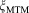 estimates indicate that the curvature generally corresponds to subexponential decay across a range of middle-high thresholds. Figure 4 shows that 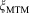 actually reflects the climatological spatial patterns as intended by *Deidda* [2010] in developing the MTM procedure. This is particularly evident focusing on United States and Australia (see also local maps in the supporting information). For example, the winter rainfall in United States is characterized by a prominent subexponential behavior in the central eastern part of the country, and a moderate subexponential behavior along the east coast, thus reflecting the clear effect of the Appalachian mountains. Similarly, for Australia, we can distinguish climate zones, such as the southwest and the east coast, which approximately reflect Köppen–Geiger classification zones (see supporting information).

**Figure 5:**
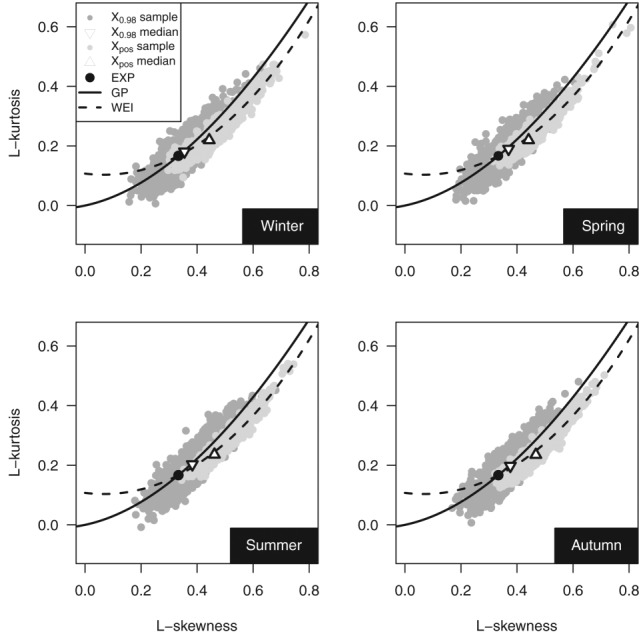
L-skewness versus L-kurtosis of POT values for the 1970–2011 sample.

[24] Going back to the overrejection of GP hypothesis, the nature of the POT daily rainfall distribution is further investigated by using the L-moment ratio diagrams (LMRDs) [*Hosking*, 1990]. Figure 5 shows the scatterplot of the empirical L-kurtosis (fourth L-moment ratio) versus L-skewness (third L-moment ratio) for the four seasons and POT values corresponding to thresholds equal to zero (denoted as “*X*_pos_” sample) and to 98th percentile thresholds (denoted as “*X*_0.98_”). The medians of the L-moment ratios and the theoretical points and curves corresponding to exponential (EXP), GP, and Weibull (WEI) distributions are also shown. The median of the L-moment ratios of *X*_0.98_ is close to the theoretical point corresponding to EXP, and the cloud is aligned along the GP curve. Therefore, LMRDs confirm the suitability of GP as a good candidate for modeling POT daily rainfall values over high thresholds. Moreover, since the points on the left (right) of the EXP point correspond to a distribution less (more) skewed than EXP, the diagram also confirms the spread of the *ξ* parameter around the zero value (Figure 3).

[25] Almost all the points corresponding to *X*_pos_ and their medians lie above and to the right of EXP, indicating that the distribution of positive daily rainfall values is generally heavy-tailed. This behavior is coherent with the results of *Deidda and Puliga* [2006] for 200 time series with at least 39 years of daily rainfall observations recorded between 1922 and 1980 in Sardinia (Italy). However, our global data set exhibits a better alignment along the WEI curve. This empirical result confirms previous findings reported in the literature. Namely, *Wilson and Toumi* [2005] showed that WEI can be derived by physical considerations of the nature of the rainfall and its stretched exponential tail explains the subexponential behavior of precipitation. Indeed, even though WEI is attracted to the Gumbel asymptote 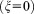, it is well known that the convergence can be very slow [*Cook and Harris*, 2004; *Papalexiou and Koutsoyiannis*, 2013], and the WEI distribution can have either an apparent heavy or bounded tail depending on the value of its shape parameter in a penultimate sense [*Cook and Harris*, 2004; *Reiss and Thomas*, 2007; *Furrer and Katz*, 2008]. In addition, *Papalexiou et al*. [2013] recognized that WEI performance in modeling POT values improves as the threshold decreases from the 95th to the 90th percentile of the nonzero values. This result is also coherent with the findings of *Papalexiou and Koutsoyiannis* [2012] concerning strictly positive rainfall values from 11,519 daily rainfall series. They showed that the generalized Gamma distribution, which can be considered as a generalization of WEI, performs better than Burr type XII, which is in turn a generalization of GP. The progressive shift from GP to WEI as the threshold decreases can therefore explain the increasing rejection of the GP hypothesis displayed in Figure 2, without excluding however the possibility that other causes may contribute. From a more operational point of view, it should be noted that WEI and GP were recognized as good candidates to model daily rainfall (the whole range and/or the upper tail) in the literature dealing with daily rainfall generators. For example, *Herr and Krzysztofowicz* [2005] and *Serinaldi* [2009] used WEI (fitted on a monthly basis) for the whole range of daily rainfall values (outperforming the exponentially tailed Gamma distribution). WEI and GP have been also used as components of hybrid models [e.g., *Vrac and Naveau*, 2007; *Furrer and Katz*, 2008; *Hundecha et al*., 2009; *Carreau and Vrac*, 2011; *Li et al*., [Bibr b47],[Bibr b48]]. This overview shows that starting from different point of views (theoretical, empirical, and operational), literature results are not so far from each other, and a suitable merging can be the basis to develop a theoretically consistent and operationally effective representation of the rainfall distribution closer to empirical observations.

## 4. Analysis of 1900–2011 Data

[26] In this section, we apply the same techniques used to analyze the 1970–2011 data set, except with some modifications required by the different rationale of the sample selection and purposes. As mentioned in section, the 1900–2011 data set has been split in subsamples of length varying from 10 to 110 years by 5 year steps. Based on the results reported in the previous section, the analysis focuses on the fixed threshold of the 98th percentile of the rainfall values. This choice guarantees to select about two events per year on a seasonal basis making the results comparable with previous studies that used a number of POT equal to the number of years. Moreover, the 98% threshold returns POT values that are reasonably GP distributed according to results reported in the previous section. Obviously, this choice is not optimal either in terms of use of the information held in the data or for at-site specific analysis, but it is a good compromise to perform a coherent study on a global data set.

[27] Similarly to the 1970–2011 case, the 1900–2011 results are given in terms of percentages of rejection. Unlike the 1970–2011 case, the subsampling procedure applied to 1900–2011 data produces a varying number of series going from 1243 10-year series to 113 110-year series. This implies that the uncertainty of the expected number of rejections varies as a result of the different number of Bernoulli trials (i.e., testing exercises). The 95% uncertainty areas around the expected nominal 5% percentage of rejection reflect this setting. Obviously, the uncertainty areas are just approximated as they do not take into account possible violations of the independence hypothesis.

**Figure 6:**
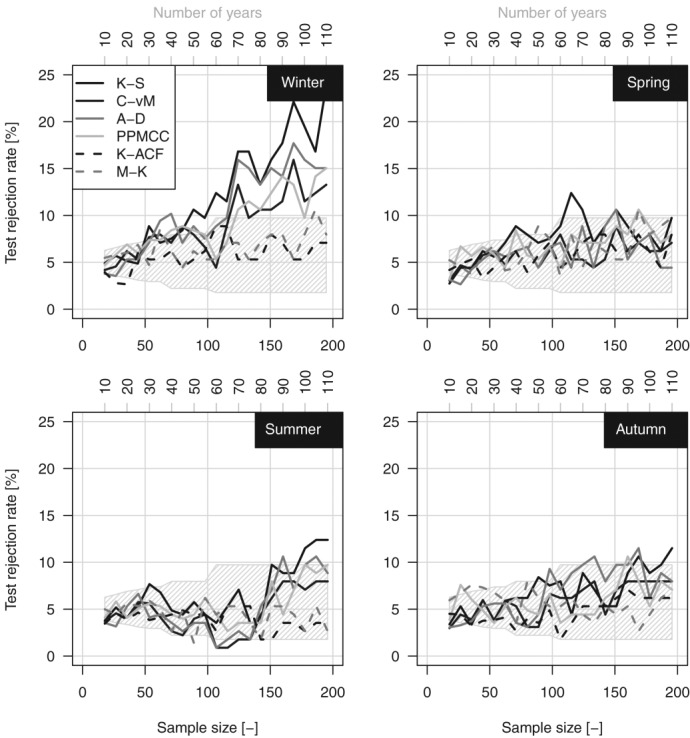
Percentage of rejection of the null hypotheses (“lag-1 K-ACF = 0,” “no monotonic trend,” and “data from GP distribution”) for the 1900–2011 sample.

**Figure 7:**
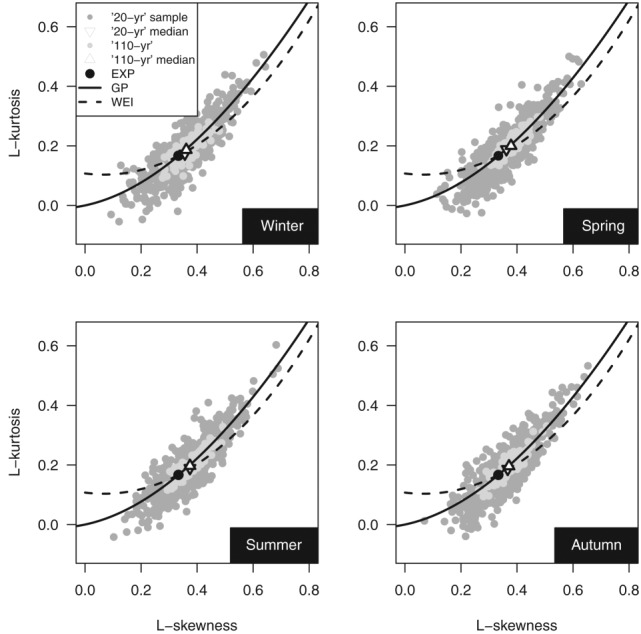
L-skewness versus L-kurtosis of POT values for the 1900–2011 sample.

[28] The K-ACF and M-K patterns in Figure 6 indicate that 98% POT values are reasonably uncorrelated and trend-free independently of length of the time series. The four goodness of fit tests highlight that the GP distribution is a good candidate to describe the 98% POT events. Overrejection (15–20%) emerges in winter for series spanning more than 60 years. The LMRDs in Figure 7 confirm that GP is able to describe the POT data independent of the record length. The mode of the empirical L-moments lies to the right of EXP, thus revealing a tendency toward a subexponential behavior. This aspect is further investigated by studying the GP *ξ* parameter.

**Figure 8:**
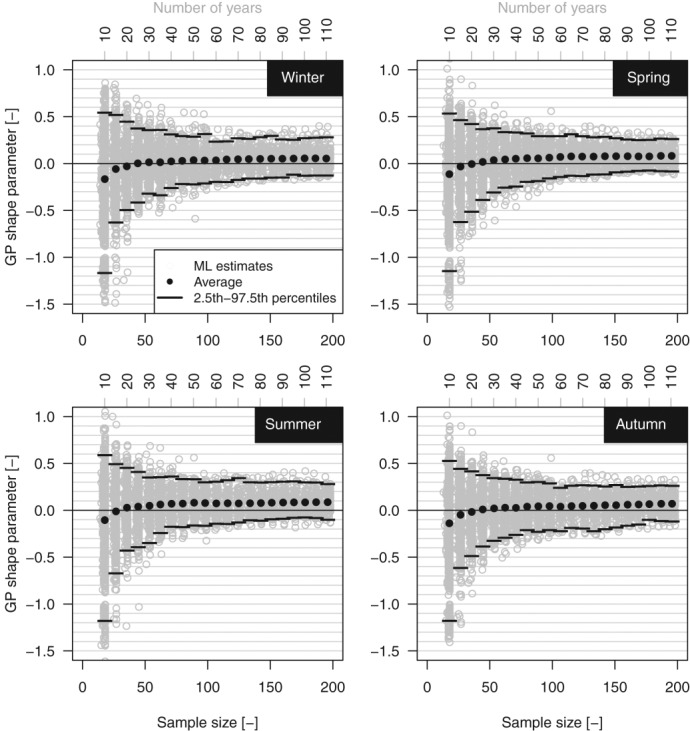
GP shape parameter versus sample size (record length) for the 1900–2011 sample.

**Figure 9:**
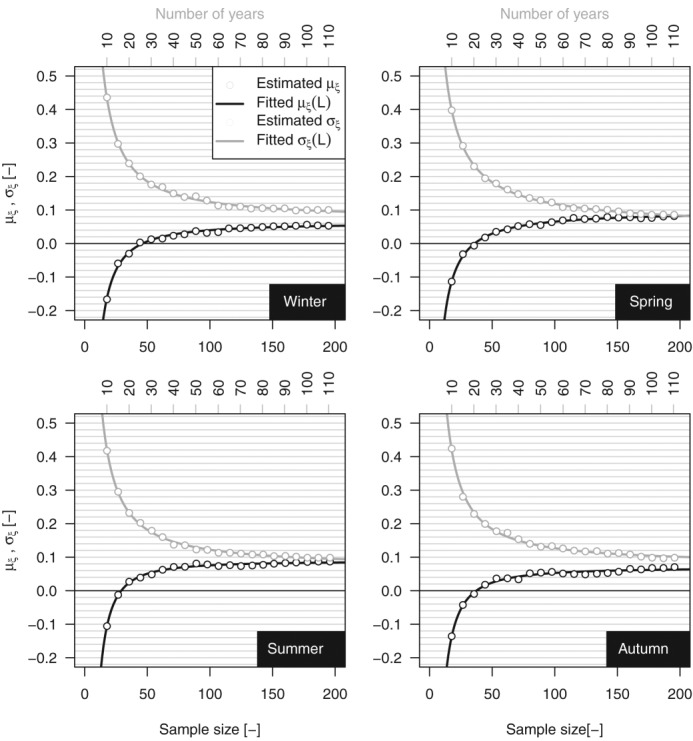
Mean and standard deviation of GP shape parameter versus sample size (record length) for the 1900–2011 sample.

[26] Following *Papalexiou and Koutsoyiannis* [2013], we have studied the relationship between *ξ* and the record length. Figure 8 is analogous to Figure 9a reported by *Papalexiou and Koutsoyiannis* [2013] for the *ξ* parameter of the GEV distribution fitted on AM values extracted from the GHCN-Daily database. In the present case, Figure 8 shows the average value of *ξ* corresponding to the average sample size obtained by selecting rainfall values exceeding the 98th percentile in subsamples with length from 10 to 110 years. We further highlight that the sampling procedure implies that several *ξ* values (positive and negative) refer to subsamples (e.g., 10 year samples) extracted from the same 110 year time series, thus reflecting the uncertainty of estimating *ξ* on short time series from a specific geographic location. As the length increases, the uncertainty decreases and the sample mean of *ξ* seems to converge to a stable and positive value. Following the same approach of *Papalexiou and Koutsoyiannis* [2013], we try to define asymptotic values for the mean and standard deviation of *ξ* and possibly an asymptotic distribution. Figure 9 displays the empirical mean and standard deviation of *ξ* for each percentile threshold and season. The empirical values are therefore fitted by a curve with equation 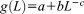 where *L* denotes the sample size and *a*, *b*, and 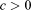 are parameters fitted by minimizing the least square error [*Papalexiou and Koutsoyiannis*, 2013]. Since 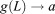 for 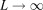, *a* describes the asymptotic limit of *g*(*L*). Figure 9 shows that the quality of fitting is remarkable. The parameters reported in Table1 indicate that the asymptotic mean value of *ξ* tends to be within the range (0.061, 0.097) which is slightly lower than the values 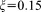 and 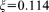 reported by *Koutsoyiannis* [2004b] and *Papalexiou and Koutsoyiannis* [2013], respectively, for the shape parameter of the GEV distribution of AM values.

**Table 1:** Seasonal Values of the Parameters of Equation (4)

Statistic	Parameter	Winter	Spring	Summer	Autumn
*μ_ξ_*(*L*)	*a_μ_* ≡ *μ_ξ_*	0.061	0.097	0.088	0.069
	*b_μ_*	−11.182	−4.812	−18.800	−13.710
	*c_μ_*	1.360	1.089	1.590	1.461
*σ_ξ_*(*L*)	*a_σ_* ≡ *σ_ξ_*	0.072	0.042	0.071	0.079
	*b_σ_*	9.039	4.605	8.497	8.946
	*c_σ_*	1.119	0.891	1.109	1.138

[30] To validate our results we repeat a Monte Carlo experiment carried out by *Papalexiou and Koutsoyiannis* [2013] and adapted to the POT context. We test the hypothesis that *ξ* follows a Gaussian distribution 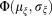 with the asymptotic values of mean and variance reported in Table1 by simulating from GP distributions with the same threshold and scale parameter values estimated on each 98% POT time series, and shape parameter sampled from 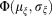. The sample size of the synthetic samples equals the actual number of POT events selected by the 98% threshold for each length (from 10 to 110 years). The GP shape parameter is therefore estimated on these simulated samples. If the “true” distribution of *ξ* is 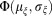, it is expected that the distribution of *ξ* for short synthetic samples reproduces that of the corresponding observed samples. In addition, the same simulation exercise has been repeated by sampling *ξ* from 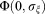 in order to show that the average asymptotic value of *ξ* is really different from zero. Results are summarized in Figure 10 as *Q-Q* plots. The almost perfect alignment along the 1:1 line denotes that the distribution of *ξ* values estimated on the samples simulated by using 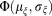 accurately reproduces the distribution of *ξ* values estimated on the observed series for every length and season. On the other hand, using 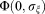 results in a systematic underestimation of *ξ*. Thus, the range of *ξ* obtained from time series of ∼50 years (a typical length for hydrologic time series) is coherent with a process which is subexponential on average and fluctuates according to 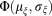, rather than with fluctuations around an exponentially decaying asymptote.

**Figure:**
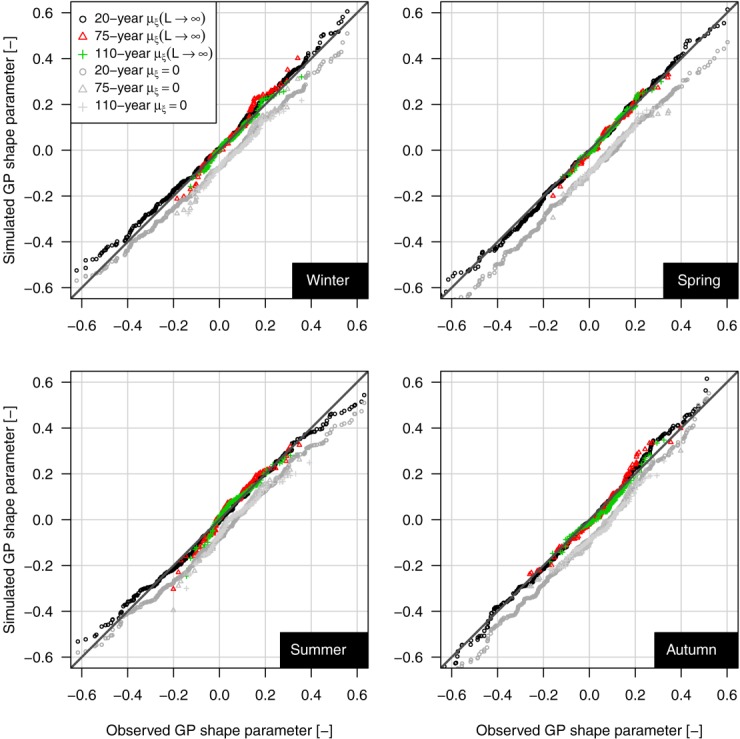
*Q-Q* plot of GP shape parameter.

[31] The causes of this behavior can be different. A reasonable explanation is based on the theory of compound distributions [e.g., *Dubey*, 1970] which are a particular case (for univariate distributions) of the more general concept of doubly stochastic processes [e.g., *Cox*, 1955; *Tjøstheim*, 1986] also known as superstatistics in statistical physics and hydroclimatology [e.g., *Beck*, 2001; *Porporato et al*., 2006]. Compound distributions are statistical models whose parameters fluctuate according to another distribution with specified parameters. The superposition of the distribution of the main random variable and the distributions of the parameters returns compound models that usually exhibit tails heavier (subexponential or power-law decaying) than the original distribution with constant parameters. Dealing with the analysis of daily rainfall extremes, *Koutsoyiannis* [2004a] explored this mechanism showing by analytical derivations and Monte Carlo simulations that fluctuations in the parameters of the parent distribution affect the tail behavior of BM making the convergence toward the Gumbel asymptote very slow. This interpretation in terms of compound distributions is coherent with the fluctuations of the climate processes over varying time scales and highlights a subtle nonstationarity whose effect can be more relevant than that related to monotonic or nonmonotonic slowly varying and detectable trends.

## 5. Bias Correction With Uncertainty

[32] Based on the above results, under the hypothesis that the true distribution of *ξ* is 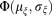 for 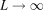, the distribution of *ξ* for a finite sample size *L* is 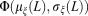, with


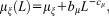
(4a)


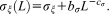
(4b)

**Figure 11:**
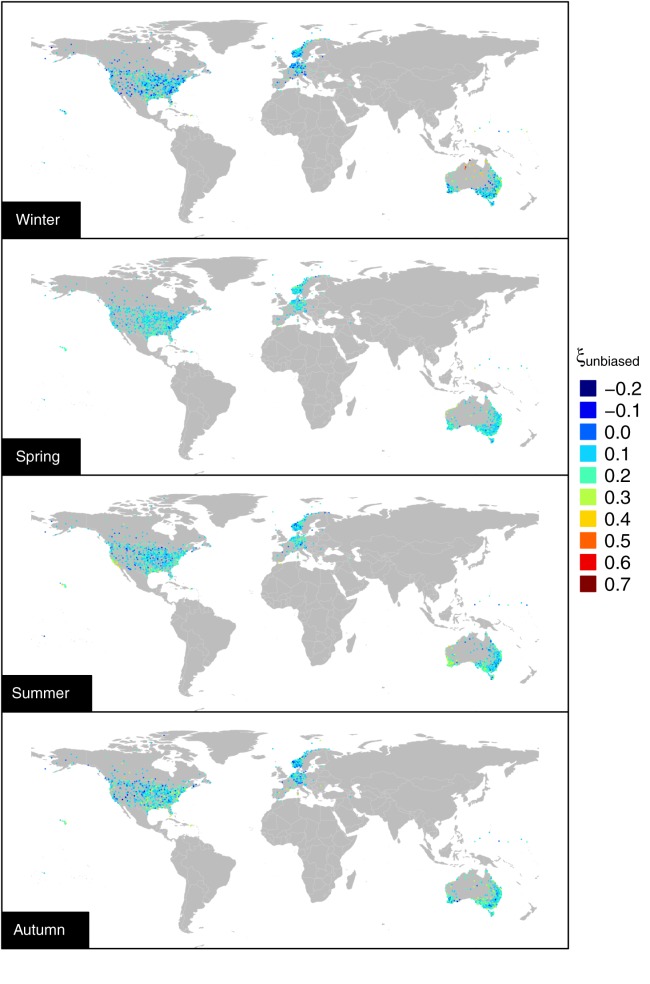
Spatial pattern of bias corrected GP shape parameter for the 1970–2011 sample (see supporting information for local maps).

[33] Using the parameters reported in Table1, we can extend to GP distribution (and 98% POT exceedances) the unbiased estimator proposed by *Papalexiou and Koutsoyiannis* [2013] for GEV:


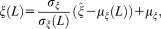
(5)

where 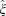 is the ML estimate of *ξ*. Equation 6 was applied to the *ξ* values computed on the 98% POT samples extracted from the 1970–2011 data set. Since equation 6 is a linear transformation, the ranks of the original estimates are preserved and also their spatial patterns (when present). Maps of the bias corrected values of *ξ* for the 98% POT exceedances are reported in Figure 11, whereas local maps for United States, Europe, and Australia are provided in the supporting information.

**Figure 12:**
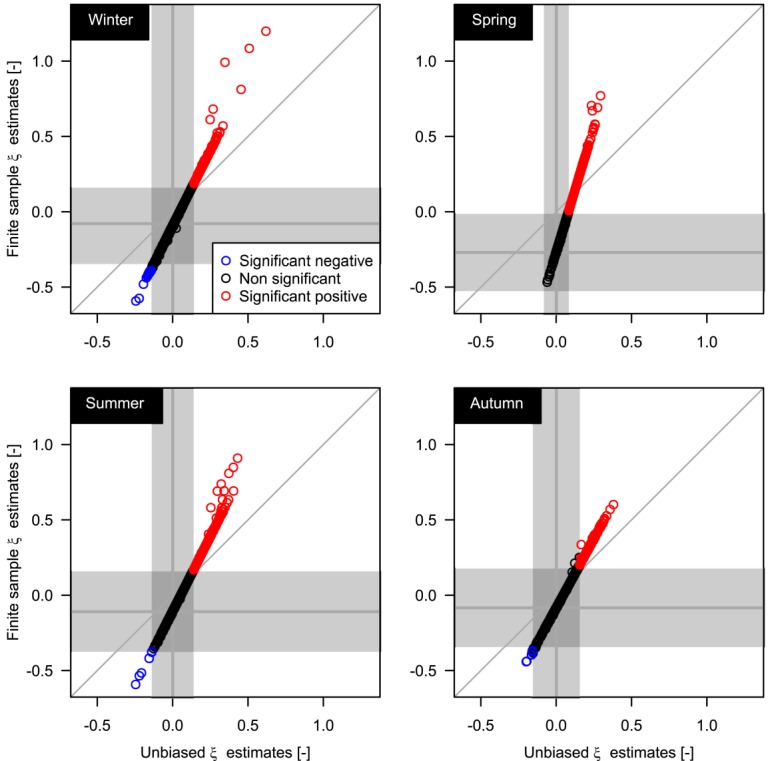
Effect of bias correction formula in equation 6. The vertical band denotes the 95% CI around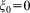 computed by the asymptotic variance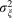, whereas the horizontal band denotes the 95% CI computed by the average variance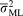 of the ML estimates. The horizontal band is centered around the *ξ* value corresponding to the asymptotic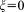.

**Figure 13:**
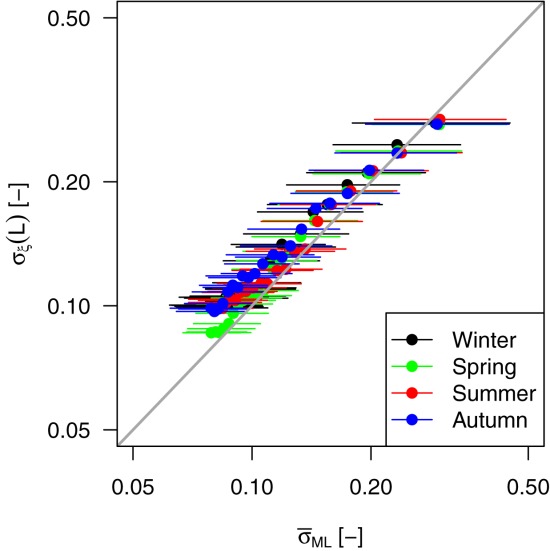
Relationships between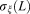 and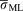 for each season. The largest (smallest) values correspond to *L* = 10 years (*L* = 110 years). Segments denote the 90% confidence intervals of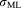.

[34] Figure 12 shows the effect of the bias correction, i.e., a shift of *ξ* values toward positive values and a variance reduction. Unlike *Papalexiou and Koutsoyiannis* [2013], the bias correction preserves a large number of negative values, thus rising the question about the upper bounded nature of rainfall, especially for specific geographic areas such as the northern Europe, where *ξ* tends to show low values. The difference between our results and those of *Papalexiou and Koutsoyiannis* [2013] can be ascribed to the smaller (larger) seasonal asymptotic averages (variances) obtained in this study for GP *ξ* values compared with asymptotic values obtained by *Papalexiou and Koutsoyiannis* [2013] for the GEV shape parameter. However, it can be shown that the difference is only apparent when the uncertainty is accounted for.

[35] A standard approach to assess the uncertainty of the ML estimates of the GP parameters is to complement the point estimates with confidence intervals (CIs) relying on the asymptotic properties of the ML estimators [*Coles*, 2001, pp. 30–33]. In particular, since the inverse of the observed information matrix gives the variance 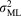 of the GP parameter estimates and the ML estimator is approximatively Gaussian distributed, the approximate 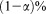 CIs for each *ξ* value in Figure 3 can be computed as 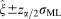, where 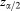 is the 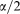 standard normal quantile. It should be noted that ML asymptotic properties hold for 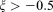 [*Coles*, 2001, pp. 54–55], and therefore, for the largest part of our estimates apart from a few cases corresponding to very short time series (see Figure 3). CIs can also be defined for the unbiased estimates as their asymptotic distribution is approximately Gaussian with variance 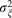.

[36] CIs can be used to highlight which estimates are significantly different from a fixed value 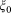 (e.g., 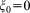) checking if the 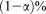 CIs include 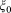 or building the 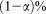 CI around 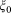 and checking which estimates fall outside the CI. The second approach has been used for the sake of easier visualization in Figure 12. The vertical band denotes the 95% CI around 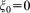 computed by the asymptotic variance 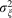, whereas the horizontal band denotes the 95% CI computed by the average variance 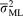 of the 1898 ML estimates. For each season, the horizontal band is centered around the *ξ* value corresponding to the asymptotic 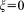 (obtained by inverting equation 6 for 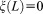). Both the methods (finite sample and asymptotic) provide CIs that cover the *ξ* values corresponding to the same set of time series (crossing area of horizontal and vertical bands) and indicate that almost all negative values of *ξ* cannot be classified as significantly different from zero (at most, only 16 values out of 1898 are recognized as negative and significant in winter). The agreement between the two methods is a relevant aspect because 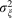 results from an independent set of time series (the 1900–2011 sample) using a particular sampling procedure, whereas 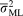 is computed by the 1898 values of 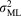 returned by ML estimation. This means that on average the ML variance related to the estimation of *ξ* on a single POT sample is close to the variance of the point estimates corresponding to a set of POT samples spread worldwide (used to obtain 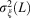 and 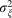). To validate this hypothesis, we plotted the values of 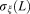 reported in Figure 9 versus the corresponding values of 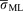. The scatterplot in Figure 13 confirms this relationship apart from a slight bias and indicates that the spatial variability of *ξ* (summarized by 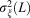) is no much different from the sampling variability (summarized by 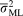). Therefore, even though *ξ* exhibits evident spatial patterns related to different climate regimes (as is shown in Figure 11 and supporting information), the amplitude of its fluctuations is coherent with the pure sampling variability. In other words, we can hypothesize a common asymptotic behavior of POT rainfall which is heavy-tailed on average and fluctuating with well-defined spatial patterns. Moreover, these results further confirm that the hypothesis of an (operational) upper bound for the rainfall distribution is not supported by empirical evidence if the sampling uncertainty is taken into account.

## 6. Conclusions

[37] In this study, the distribution of daily rainfall values exceeding fixed thresholds have been explored by using a worldwide data set and accounting for the role exerted by record length and the threshold values. The analysis has been performed within the framework of classical EVT, complementing the study of the upper tail behavior with a preliminary analysis for temporal correlation and monotonic trends of POT data.

[38] The analysis of 1898 time series spanning from 1970 to 2011 revealed that there is no evidence for time correlation and monotonic trends in POT data for every threshold ranging from the 95th to 99.5th percentiles and all seasons. Indeed, for these data, the percentage of rejection of the null hypothesis has been found close to the nominal value (here, 5%). These results are valid under the hypothesis that the POT events are spatially uncorrelated. However, since spatial correlation inflates the variance of the test statistics under the null hypothesis, our findings are expected to be even more evident if the effect of spatial correlation is accounted for.

[39] Based on these results, the data were deemed suitable to be studied in the framework of the classical stationary EVT. Four goodness of fit tests indicated that the percentage of rejection of GP distribution is close to the nominal 5% only for high thresholds (>98%), for which the POT sample size becomes very small (less than two events per year on a seasonal basis), the uncertainty increases and the power of discrimination of the tests might be low. As the threshold decreases, the EVT assumptions that justify the GP asymptote are progressively less valid and the rejection rate increases. Indeed, the LMRDs showed that the distribution of POT values evolves from GP to WEI coherently with the theory of the penultimate asymptotic distribution. This explains the results returned by the goodness of fit tests. However, beyond formal statistical tests and LMRDs, distinguishing between GP and WEI is not straightforward, and GP may outperform WEI in some cases and vice versa based on the performance criterion adopted. Moreover, it should be noted that rounding off effects and undetected (and often undetectable) measurement errors may influence the results. Therefore, even though these effects are not always easy to quantify, especially in large data sets collected by different offices using heterogeneous methods, their presence must be considered as a possible source of uncertainty in this kind of analyses.

[40] The average value of the GP shape parameter *ξ* increases and becomes positive as the threshold decreases (including more and more data in the POT sample), whereas the variability decreases. The behavior of *ξ* in the virtual limit condition of a zero threshold has been studied by using the maximum likelihood MTM proposed by *Deidda* [2010] based on the expression of the complete discrete-continuous distributions of all rainfall values, under the working hypothesis that GP is a suitable model over the entire range of positive rainfall records. As mentioned in section, even though this hypothesis contradicts the empirical evidence in several cases, it was used to explore the curvature of the upper part of the rainfall distribution using the sign of *ξ* as a measure, and it does not imply that GP (with constant parameters) is a suitable model for the whole range of rainfall values. When the threshold is high and the number of POT exceedances is small, *ξ* is highly volatile, fluctuates around zero, and is affected by high uncertainty, thus pointing to (an apparent) general exponential behavior of the upper tail. On the other hand, MTM results indicate that *ξ* is almost always positive when GP is fitted on the middle and upper part of the rainfall distribution, and the parameter fluctuations due to the threshold selection are smoothed out by MTM. Therefore, the large fluctuation of *ξ* (reported in the literature and in this study) corresponding to a high threshold is coherent with a general heavy tail behavior of the rainfall process, when we consider the middle-high rainfall values.

[41] The analysis of 113 time series spanning from 1900 to 2011 allowed us to explore the effect of the record length (from 10 to 110 years) for a fixed threshold (here the 98th percentile). As for the 1970–2011 sample, the POT values were tested for temporal dependence, monotonic trends, and distribution. The percentage of rejection of all the tests is coherent with the nominal significance level (5%) for all records, once the uncertainty of the multiple testing exercise is accounted for. LMRDs confirm that GP distribution is a suitable candidate independent of the record length.

[42] The average value of GP shape parameter increases and tends to a positive value as the record length increases, whereas the variability decreases. Under the hypothesis of the existence of an asymptotic distribution for the shape parameter, it has been shown that the apparent exponential decay of the upper tail of the rainfall distribution observed in short time series is coherent with an asymptotic process which fluctuates around an average heavy tail behavior, whereas fluctuations around an asymptotic exponential decay return biased results. Based on the duality of GP distribution for exceedances and GEV distribution for block maxima, our results confirm and strengthen the previous findings of *Papalexiou and Koutsoyiannis* [2013] based on the AM analysis and further support the hypothesis that the heavy tail behavior of extreme rainfall is coherent with fluctuations of the parent distribution. These fluctuations can result from two main causes: (1) the mixture of different processes, namely, extreme and nonextreme observations selected via threshold values that are not high enough; and (2) the temporal fluctuations of the parent distribution and/or its parameters, which evolve over long time scales and are difficult to detect in the commonly available short time series. While we tend to limit the weight of the first cause based on our threshold analysis, the latter is coherent with the fluctuation of climate and other physical mechanisms driving the rainfall process.

[43] From a practical point of view, we have extended the bias correction formulae provided by *Papalexiou and Koutsoyiannis* [2013] for the GEV shape parameter to GP shape parameter corresponding to POT values over the 98th percentile threshold on a seasonal basis. Applying this correction to the *ξ* estimates corresponding to the 1970–2011 data set and introducing finite sample and asymptotic confidence intervals, we have shown that negative values of *ξ* are not statistically significant, thus confirming the hypothesis of an asymptotic subexponential behavior on average. The uncertainty analysis also revealed an almost exact correspondence between the sampling variance of the maximum likelihood estimates and the spatial variance. This result further supports the hypothesis that POT extreme rainfall follows a common asymptotic behavior whose fluctuations have an amplitude coherent with random fluctuations but cluster in the space according to the different climate regimes.
